# Temperature elevation in the human brain and skin with thermoregulation during exposure to RF energy

**DOI:** 10.1186/s12938-017-0432-x

**Published:** 2018-01-08

**Authors:** Sachiko Kodera, Jose Gomez-Tames, Akimasa Hirata

**Affiliations:** Department of Electrical and Mechanical Engineering, Nagoya Institute of Technology, Nagoya, 466-8555 Japan

**Keywords:** Human safety, Safety guidelines, Computational dosimetry, Vasodilation, Bioheat equation

## Abstract

**Background:**

Two international guidelines/standards for human protection from electromagnetic fields define the specific absorption rate (SAR) averaged over 10 g of tissue as a metric for protection against localized radio frequency field exposure due to portable devices operating below 3–10 GHz. Temperature elevation is suggested to be a dominant effect for exposure at frequencies higher than 100 kHz. No previous studies have evaluated temperature elevation in the human head for local exposure considering thermoregulation. This study aims to discuss the temperature elevation in a human head model considering vasodilation, to discuss the conservativeness of the current limit.

**Methods:**

This study computes the temperature elevations in an anatomical human head model exposed to radiation from a dipole antenna and truncated plane waves at 300 MHz–10GHz. The SARs in the human model are first computed using a finite-difference time-domain method. The temperature elevation is calculated by solving the bioheat transfer equation by considering the thermoregulation that simulates the vasodilation.

**Results:**

The maximum temperature elevation in the brain appeared around its periphery. At exposures with higher intensity, the temperature elevation became larger and reached around 40 °C at the peak SAR of 100 W/kg, and became lower at higher frequencies. The temperature elevation in the brain at the current limit of 10 W/kg is at most 0.93 °C. The effect of vasodilation became notable for tissue temperature elevations higher than 1–2 °C and for an SAR of 10 W/kg. The temperature at the periphery was below the basal brain temperature (37 °C).

**Conclusions:**

The temperature elevation under the current guideline for occupational exposure is within the ranges of brain temperature variability for environmental changes in daily life. The effect of vasodilation is significant, especially at higher frequencies where skin temperature elevation is dominant.

## Background

There are two international guidelines [1, 2] for human protection from electromagnetic fields, which have been set forth by the World Health Organization (WHO). In the guidelines, the metric for human protection is the specific absorption rate (SAR) averaged over 10 g of tissue for localized exposure to electromagnetic fields with frequencies ranging from 100 kHz to 3 GHz [2] or 10 GHz [1]. The International Commission on Non-Ionizing Radiation Protection (ICNIRP) and IEEE have been discussing revisions to the new guideline, including revisions on the averaging volume and transition frequency of SAR.

In the current ICNIRP guidelines [1], the averaging volume of SAR corresponds to contiguous tissue, while the IEEE standard [2] specifies a cube. The limit is 10 W/kg for occupational exposure or in restricted areas, and a reduction factor of 5 is applied for general public general public or in unrestricted areas. SAR averaging over contiguous tissue is feasible in computation, but is not practically feasible as later noted by the ICNIRP [3]. Thus, a cubic averaging volume is used in the international standard for product safety when ensuring product compliance [4, 5]. Considering the 1998 ICNIRP guidelines [1], the compliance procedure at frequencies from 6 to 10 GHz has been discussed in the IEC Technical Committee 106.

The averaging mass of the SAR has been discussed by different research groups (e.g., [6–8]). The main conclusion drawn by international experts is that the averaging mass of 10 g, corresponding to a cube with a side length of approximately 22 mm, is a good metric to correlate with the local temperature rise [9]. A few studies discussed the rationale of the limit for an occupational exposure of 10 W/kg.

The point SAR measurement and local temperature elevation in anesthetized rabbit eyes has been cited in the ICNIRP guidelines [1]. The SAR threshold value estimated with a probe finite dimension was 137 W/kg [10]. The temperature elevation computed in the brain is listed on Table C.2 in Annex C of the IEEE standard C95.1 [2]. The temperature elevation at a peak 10-g SAR is approximately 1–2 °C. In the IEEE standard [2], the temperature elevation in the eye without anesthesia has been reported to be smaller [11]. Later, Hirata reported that this is caused by the effect of thermoregulation in non-anesthetized rabbits [12]. No computational study listed therein considered the effect of thermoregulation, which is inherent in homeotherms such as humans, on the human temperature elevation for localized exposures.

After the publication of IEEE C95.1 [2], the authors investigated temperature elevations in rat brains due to localized radio-frequency exposure. We demonstrated that the temperature elevation in heat-sensitive tissues of small animals becomes smaller than that when thermoregulation is ignored [12, 13]. No previous studies evaluated the temperature elevation in human head models for local exposure while considering thermoregulation. It should be noted that several studies computed the temperature elevation for whole-body exposures [14–18].

The abovementioned limit is intended to prevent excessive heating, especially in internal tissue (e.g., brain and eye). The heating factor, which is defined as the ratio of temperature elevation to the SAR, was introduced and discussed extensively [19]. After obtaining this factor, temperature elevation can be estimated in the regime where thermoregulation can be neglected. As one of the drawbacks of this concept, the temperature under thermoneutral conditions depends on the body part and is not directly related to thermal damage. For example, the information of the location where the maximum temperature appeared is missed. When thermoregulation is ignored, the heating factor value becomes conservative when the tissue temperature becomes large enough to activate thermoregulation. Thermoregulation, especially for vasodilation, can move heat away and maintain homeostasis.

Many researchers developed a thermoregulatory response model that included vasodilation. These models are based on the measurements of internal tissues during animal studies. Some of these studies used an invasive approach with anesthesia (e.g., [20]). Even though a limited amount of data on thermoregulation is available for internal tissues in humans, it is known that thermoregulation in humans is superior to that of other species [21]. In this study, we investigate brain temperature elevation for excessive SAR exposure levels to find out how vasodilatation can affect the temperature elevation and how it relates to the exposure limits.

## Methods

Our bioheat modeling has been mentioned in our previous studies [22]. The vasodilation model was coupled with the bioheat modeling. Vasodilation modeling of the brain and skin was based on local exposure measurements in rats [23] and cutaneous veins of a dog with local warming [24], respectively. Our computational model has been validated for different exposure scenarios including the partial-body exposure [25], where human legs are immersed in hot water [22].

### SAR computation

A finite-difference time-domain (FDTD) method was used to calculate the absorbed microwave power [26]. The SAR is defined as

1 $$SAR({\mathbf{r}}) = \frac{{\sigma ({\mathbf{r}})}}{{2\rho ({\mathbf{r}})}}\left| {{\mathbf{E}}({\mathbf{r}})} \right|^{2} ,$$

where |**E**(**r**)| denotes the peak value of the electric field at position **r**. The parameters *σ* and *ρ* denote the conductivity and mass density of the tissue, respectively. The dielectric properties of each tissue type were determined with a 4-Cole–Cole dispersion model [27]. Convolutional perfectly matched layer absorbing boundary conditions was used for absorbing outgoing scattered waves to simulate an infinite space [28].

To calculate the spatially averaged SAR over 10 g of tissue, contiguous tissue and 10 g of tissues in a cube shape were used as specified by the ICNIRP guidelines [1] and the IEEE C95.3-2002 standard [5], respectively. In [1], no detailed algorithm has been prescribed. Thus, the algorithm we developed in [29] is used in this study.

### Temperature computation

The Pennes bioheat transfer equation considers heat exchange mechanisms, including heat conduction, blood perfusion, and resistive heating, as follows [30]:

2 $$C\left( {\mathbf{r}} \right)\rho \left( {\mathbf{r}} \right)\frac{{\partial T\left( {{\mathbf{r}},t} \right)}}{\partial t} = \nabla \cdot \left( {K\left( {\mathbf{r}} \right)\nabla T\left( {{\mathbf{r}},t} \right)} \right) \,+\, \rho \left( {\mathbf{r}} \right)SAR\left( {\mathbf{r}} \right) \,+ \,A\left( {{\mathbf{r}},t} \right) \,-\, B\left( {{\mathbf{r}},t} \right)\left( {T\left( {{\mathbf{r}},t} \right) \,- \,T_{B} \left( {{\mathbf{r}},t} \right)} \right),$$

where **r** and *t* denote the position vectors of tissue and time, respectively, and *T*(**r**, *t*) and *T*_*B*_(**r**, *t*) denote the temperatures of tissue and blood, respectively. In addition, *C* denotes the specific heat of the tissue, *K* denotes the thermal conductivity of the tissue, *A*(**r**, *t*) denotes the metabolic heat, and *B*(**r**, *t*) denotes the factor related to blood perfusion. The SAR(**r**, *t*) determined from Eq. 1 is substituted into the bioheat equation as a heat source.

The boundary condition between tissues and external air is

3 $$- K\left( {\mathbf{r}} \right)\frac{{\partial T\left( {{\mathbf{r}},t} \right)}}{\partial n} = H\left( {\mathbf{r}} \right) \cdot \left( {T\left( {{\mathbf{r}},t} \right) - T_{a} } \right) + EV({\mathbf{r}},t),$$

where *T*_*a*_ (= 27 °C), *H*, and *n* denote the ambient temperature, heat transfer coefficient, and a vector normal to the body surface, respectively. *EV* denotes the evaporative heat loss function.

The volume-averaged blood temperature is changed such that the first law of thermodynamics is satisfied. The change in blood temperature is defined as [14]

4 $$T_{B} \left( t \right) = T_{B0} + \int_{t} {\frac{{Q_{BT} \left( t \right) - Q_{BT} \left( 0 \right)}}{{C_{B} \rho_{B} V_{B} }}dt} ,$$

5 $$Q_{BT} \left( t \right) = \int_{V} {B\left( t \right)\left( {T_{B} \left( t \right) - T\left( {{\mathbf{r}},t} \right)} \right)dV}$$

where *Q*_*BT*_(*t*) denotes the total heat transferred from tissues to blood. In addition, *C*_*B*_ = 4000 J/(kg  °C), *ρ*_*B*_ = 1058 kg/m^3^, *T*_*B0*_, and *V*_*B*_ denote the specific heat of blood, mass density of blood, initial blood temperature, and total volume of blood, respectively. The blood temperature *T*_*B*_(**r**, *t*) was fixed at 37 °C in this study (see subsection “Computational setup of thermoregulatory modeling”).

The blood perfusion parameter *B* in skin depends on both the hypothalamus temperature and local skin temperature [24]. Skin blood perfusion is regulated by the hypothalamus temperature. The average skin temperature can be expressed as

6 $$B({\mathbf{r}},t) = \left[ {B_{0} ({\mathbf{r}}) + F_{HB} \Delta T_{H} (t) + F_{SB} \Delta T_{S} } \right] \cdot 2^{{{{\left( {T({\mathbf{r}},t) - T_{0} ({\mathbf{r}})} \right)} \mathord{\left/ {\vphantom {{\left( {T({\mathbf{r}},t) - T_{0} ({\mathbf{r}})} \right)} 6}} \right. \kern-0pt} 6}}} ,$$

where *B*_*0*_ denotes the basal blood perfusion of each tissue, Δ*T*_*S*_(*t*) denotes the average skin temperature elevation, *S* denotes the skin area, and Δ*T*_*H*_(*t*) denotes the core (hypothalamus) temperature elevation. *F*_*HB*_ = 17,500 W/(m^3^ °C) and *F*_*SB*_ = 1100 W/(m^3^ °C) also correspond to coefficients for determining the changes in the blood perfusion characteristics over time [31].

Blood perfusion in the brain is influenced by the core and brain local temperature elevations and is expressed as [13]

7 $$B\left( {{\mathbf{r}},t} \right) = B_{0} \left( {\mathbf{r}} \right) \cdot \left( {1 + F_{HB} \cdot \Delta T_{H} \left( t \right)} \right) \cdot 2^{{\Delta T\left( {{\mathbf{r}},t} \right)/F_{BB} }} ,$$

where *F*_*HB*_ (= 0.053 °C^−1^) and *F*_*BB*_ (= 13.9 °C) denote weighting coefficients relating to the variations in the core and brain temperature elevations, respectively [23]. Note that the terms associated with core temperature elevation in (4) and (5) do not affect the results in this study because core temperature elevation is small enough for localized exposure, as will be discussed in “Results” section.

Blood perfusion regulation in tissues (except for the skin and brain) is defined as [32, 33]

8 $$\begin{array}{*{20}l} {B\left( {{\mathbf{r}},t} \right) = B_{0} ({\mathbf{r}}),} & \quad {T\left( {{\mathbf{r}},t} \right) \le 39{}^{ \circ }{\text{C}}} \\ {B\left( {{\mathbf{r}},t} \right) = B_{0} ({\mathbf{r}})\left[ {1 + S_{B} \left( {T\left( {{\mathbf{r}},t} \right) - 39} \right)} \right],} & \quad {39{}^{ \circ }{\text{C}} \le T\left( {{\mathbf{r}},t} \right) \le 44{}^{ \circ }{\text{C}}} \\ {B\left( {{\mathbf{r}},t} \right) = B_{0} ({\mathbf{r}})\left[ {1 + 5 \cdot S_{B} } \right],} & \quad {44{}^{ \circ }{\text{C}} \le T\left( {{\mathbf{r}},t} \right),} \\ \end{array}$$

where *B*_0_(*r*) is based on the blood perfusion in each tissue, and *S*_*B*_ (= 0.8 °C^−1^) denotes a coefficient defining changes in the blood perfusion characteristics over time.

The thermal parameters used in the present study were the same as those in [34]. The heat transfer coefficient *H* was 8 W/(m^2^ °C) between the skin and air, and 20 W/(m^2^ °C) between the eye and air [35].

Evaporative heat loss is assumed to depend on the temperature elevation on the skin and in the hypothalamus. This relationship is defined as [36]

9 $$EV({\mathbf{r}},t) = \left\{ {W_{S} ({\mathbf{r}},t)\Delta T_{S} (t) + W_{H} ({\mathbf{r}},t)\Delta T_{H} (t) + PI} \right\} \cdot F_{EV} /S,$$

10 $$W_{S} ({\mathbf{r}},t) = \alpha_{11} \tanh (\beta_{11} \Delta T_{S} (t) - \beta_{10} ) + \alpha_{10} ,$$

11 $$W_{H} ({\mathbf{r}},t) = \alpha_{21} \tanh (\beta_{21} \Delta T_{H} (t) - \beta_{20} ) + \alpha_{20} ,$$

where *F*_*EV*_ (= 40.6 W min/g) is a conversion coefficient, and *S* is the surface area of the human body. The parameter of *PI* (= 0.63 g/min) is insensible water loss, and the coefficients are defined as *α*_10_ = 1.20 g/(min  °C), *α*_11_ = 0.80 g/(min  °C), *β*_10_ = 0.19, *β*_11_ = 0.59 °C^−1^, *α*_20_ = 6.30 g/(min  °C), *α*_21_ = 5.70 g/(min  °C), *β*_20_ = 1.03, and *β*_21_ = 1.98 °C^−1^.

Core temperature elevation is one of the essential parameters in sweating. For the maximum output power of the dipole antenna, corresponding to an SAR of 100 W/kg, the total power absorbed in by the human body is less than 30.3 W, which is a quarter of the basal metabolism of the human. Note that a whole-body averaged SAR of 4–6 W/kg (260–390 W for a 65-kg adult) results in a core temperature elevation of 1 °C. As will be discussed in the “Results” section, the effect of core temperature elevation on sweating is not dominant for the scenarios considered here.

### Numerical human head model

A realistic anatomical model of a Japanese adult male (TARO) [37] was considered. This computational anatomical model had a resolution of 2 mm. The model was segmented into 51 anatomical regions, such as the skin, muscle, bone, and brain. The head model was truncated at the bottom of the neck.

A major source of computational error in FDTD methods is discretization error. In electromagnetic simulations, this depends on the ratio between the cell size and the wavelength in biological tissue. A well-known rule for suppressing numerical dispersion error in FDTD simulations is that the maximum cell size should be smaller than one-tenth of the wavelength. Therefore, the model resolution was divided into 0.5 mm cells to satisfy this criterion at 10 GHz, which is the highest frequency used herein.

**Fig. 1:**
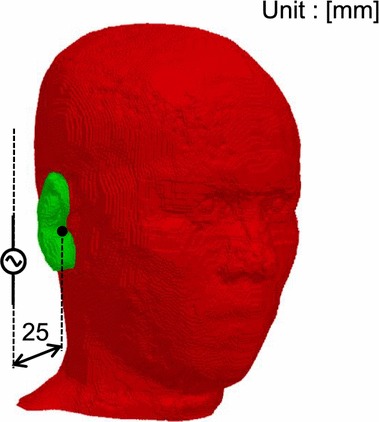
Exposure scenarios using the head part of the numeric Japanese male model

Figure 1 illustrates the exposure scenarios. As shown in Fig. 1, the separation between the dipole antenna and the surface of the head model, excluding the pinna, was set to 25 mm (14–16 mm from the pinna). For comparison, a vertically polarized truncated plane wave incident from the side of the head was also calculated. The frequencies considered in this study were 300 MHz–10 GHz. The length of the antenna was set to be equal to half the wavelength in each scenario. For comparison, the output power of the antenna was adjusted so that the 10 g averaged SAR was 2 (limited for general public and unrestricted environments), 10 (limited for restricted environments or occupational exposures), 50, and 100 W/kg. The SAR was averaged in a cubic shape following the IEEE standard [5]. Even though the SAR is not used at frequencies above 6 GHz according to IEEE [2], the same metric is used even at 10 GHz to facilitate a proper comparison.

A vertically polarized truncated plane wave, as a far-field source, was also considered for comparison. The effect of polarization is marginal at frequencies higher than the GHz region, where the penetration depth is a few centimetres or less. Temperature simulations were run for 65 min.

### Heating factor in the brain

The heating factor is an approximation that expresses the temperature elevation due to microwave exposure, as follows [38–40]:

12 $$\alpha = {\raise0.7ex\hbox{${\Delta T_{brain} }$} \!\mathord{\left/ {\vphantom {{\Delta T_{brain} } {SAR_{head} }}}\right.\kern-0pt} \!\lower0.7ex\hbox{${SAR_{head} }$}},$$

where *α* [°C kg/W] denotes the heating factor, △*T*_*brain*_ [°C] denotes the maximum temperature elevation in the brain, and *SAR*_*head*_ [W/kg] denotes the peak SAR in the head. Note that several heating factors have been introduced for different locations of peak SAR and peak temperature elevation [38].

## Results

### SAR distribution

**Fig. 2:**
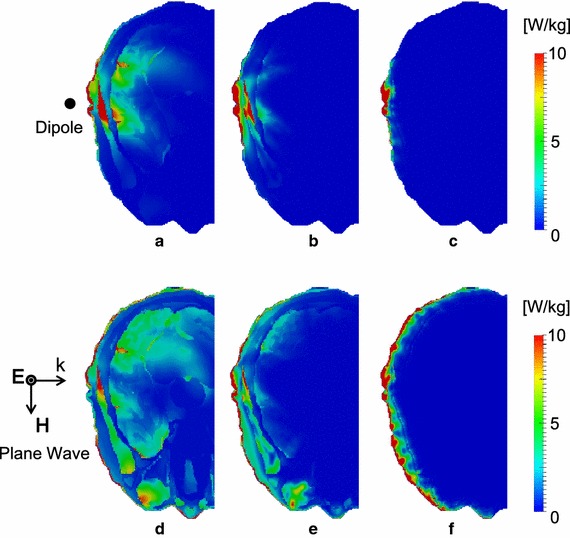
SAR distributions for each exposure scenario. The exposure from a dipole antenna at **a** 1 GHz, **b** 3 GHz, and **c** 10 GHz. Exposure from a truncated plane wave at **d** 1 GHz, **e** 3 GHz, and **f** 10 GHz. The output power was adjusted so that the peak 10 g average SAR becomes 10 W/kg

Figure 2 shows the SAR distribution from the dipole antenna and truncated plane wave at 1, 3, and 10 GHz, respectively. The reason for choosing three frequencies will be given below. As shown in the figures, the distribution of SAR concentrates on the surface layer as the frequency increases or as the penetration depth decreases. The penetration depth into muscle is 40.7 mm at 1 GHz and 3.3 mm at 10 GHz [41]. The penetration depths of the dipole antenna and the truncated plane wave are nearly the same. The exposure from truncated plane waves has a wider area than that from the dipole antenna. These tendencies are most evident, at 1 GHz.

Table 1 shows the peak SAR averaged over the cubic and contiguous regions. The average SAR over a contiguous region was larger than in a cube shape for both the dipole antenna and truncated plane wave exposure. For comparison, the dipole antenna output power was chosen as 1 W, and the truncated plane wave incident power density was 100 W/m^2^. As seen from the table, the differences between SARs calculated from the different algorithms are obvious at higher frequencies. Unlike the ICNIRP guidelines, the IEEE standards treat the pinna as the extremity. For the dipole antenna in close proximity to the pinna, most of the power is absorbed in the pinna [38]. A more detailed discussion on the effect of the averaging algorithm on the relation between SAR and temperature elevation can be found in our previous study [38]. The temperature elevation for a contiguous tissue can be estimated from the results presented in the table. As mentioned above, the cubic shape is commonly used and thus in the following discussion the SAR is averaged over a cubic shape.

**Table 1:** Computed specific absorption rate (SAR) averaged over 10 g using two averaging algorithms prescribed in the IEEE standard and ICNIRP guidelines

	300 MHz	1 GHz	3 GHz	6 GHz	10 GHz
Dipole antenna					
Cube	0.75	2.59	3.20	2.67	3.83
Contiguous	1.02	6.04	10.82	8.30	12.70
Plane wave					
Cube	8.07	4.43	6.18	4.66	4.15
Contiguous	13.34	7.85	14.15	25.77	23.80

The output power from the dipole antenna is 1 W. The incident power density of the truncated plane wave is 100 W/m^2^.

### Computational setup of thermoregulatory modeling

The initial temperature distribution *T*_0_ was determined using Eqs. 2 and 3 with *SAR* = 0 W/kg. Then, for the maximum exposure level in this study (10 g averaged SAR = 100 W/kg), the blood perfusion rate and sweating were considered in thermoregulation to compute the elevated temperature in the whole-body model for dipole antenna exposure at 1 GHz. The averaged skin and core temperature elevation (whole-body) were at most 0.3 and 0.1 °C, respectively, over 1 h duration, which is sufficient to reach the thermal steady state. The maximum temperature elevation difference in the skin between these two scenarios was 5% (11.3 and 11.9 °C in the whole-body model considering thermoregulation with sweating and in the truncated head model considering thermoregulation but without sweating and core temperature change, respectively).

These results suggest that the temperature elevation computation in the head model without considering sweating was sufficient for computing the local temperature elevation and vasodilation for local exposures. Thus, in the following discussion, the blood temperature *T*_*B*_(**r**, *t*) was fixed at 37 °C and sweating was ignored. In addition, the truncated head model was used.

### Temperature elevation and blood perfusion rate in the skin and brain

Tables 2 and 3 show the maximum temperature elevations in the brain for dipole antenna and truncated plane wave exposure, respectively. It took 64, 34, 44, 45, 49 min at 0.3, 1, 3, 6, 10 GHz, respectively to the thermal steady-state. The time needed for skin to reach the steady state was shorter than that for the brain. This occurs because of the heat conduction from the surface to the brain. The time needed to reach the steady state becomes shorter as the peak averaged SAR increased. This is because the blood perfusion effect becomes obvious, resulting in shorter thermal time constant.

**Table 2:** Spatial maximum (voxel) temperature elevation in the brain due to exposure from the dipole antenna

Peak SAR	2 W/kg		10 W/kg		50 W/kg		100 W/kg	
	T_brain_ [°C]	ΔT [°C]	T_brain_ [°C]	ΔT [°C]	T_brain_ [°C]	ΔT [°C]	T_brain_ [°C]	ΔT [°C]
300 MHz	36.77	+ 0.17	37.48	+ 0.87	39.80	+ 3.19	41.62	+ 4.76
1 GHz	36.75	+ 0.18	37.49	+ 0.93	40.00	+ 3.46	41.67	+ 5.14
3 GHz	36.71	+ 0.14	37.30	+ 0.73	39.21	+ 2.64	40.40	+ 3.83
6 GHz	36.66	+ 0.09	37.04	+ 0.46	38.32	+ 1.74	39.19	+ 2.61
10 GHz	36.66	+ 0.07	36.85	+ 0.37	37.79	+ 1.23	38.26	+ 1.70

Temperature computations including thermoregulation. The SAR was averaged over a cubic shape following the IEEE standard

**Table 3:** Spatial maximum (voxel) temperature elevation in the brain for exposure from the truncated plane wave

Peak SAR	2 W/kg		10 W/kg		50 W/kg		100 W/kg	
	T_brain_ [°C]	ΔT [°C]	T_brain_ [°C]	ΔT [°C]	T_brain_ [°C]	ΔT [°C]	T_brain_ [°C]	ΔT [°C]
300 MHz	37.22	+ 0.16	37.89	+ 0.83	40.16	+ 3.08	41.85	+ 4.79
1 GHz	36.76	+ 0.14	37.38	+ 0.76	39.90	+ 3.28	42.07	+ 5.25
3 GHz	36.81	+ 0.13	37.36	+ 0.68	39.34	+ 2.75	40.56	+ 3.97
6 GHz	36.74	+ 0.15	37.24	+ 0.75	39.35	+ 2.86	40.55	+ 4.06
10 GHz	36.61	+ 0.14	37.21	+ 0.68	38.90	+ 2.46	40.00	+ 3.52

Temperature computations including thermoregulation. The SAR was averaged over a cubic shape following the IEEE standard

The maximum SAR value appeared in the skin of the head surface at frequencies ranging from 1 to 10 GHz and in fat at 0.3 GHz. The maximum temperature elevation presented in the head appeared in the skin at frequencies of 3–10 GHz while in the muscle at 0.3 and 1 GHz. The maximum temperature elevation in the brain appeared in gray matter for all the frequencies. However, their points did not coincide with each other because of the complicated SAR distribution.

For peak SAR equal to 100 W/kg, the differences between the exposure from the dipole antenna and the truncated plane wave were 0.6, 2.1, 13.8, 55.5 and 107.5% at 0.3, 1, 3, 6 and 10 GHz. For SAR averaged over a contiguous tissue, the maximum temperature elevations in the brain were 0.45 and 0.11 °C at 1 and 10 GHz, respectively. These values occur at a peak SAR of 10 W/kg. These values were a half to one-third of the SAR averaged over a cube. The main reason for the SAR difference is attributable to the inclusion of the pinna in the averaging volume.

**Fig. 3:**
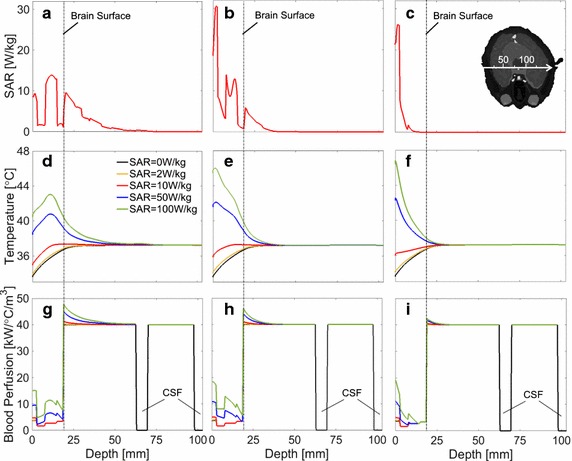
Distributions of SAR, temperature, and blood perfusion rate from the dipole antenna. The axis passes through the cross section center in Fig. 2. SAR at **a** 1 GHz, **b** 3 GHz, and **c** 10 GHz. Temperature elevation at **d** 1 GHz, **e** 3 GHz, and **f** 10 GHz. Blood perfusion rate at **g** 1 GHz, **h** 3 GHz, and **i** 10 GHz. The output power was normalized so that peak SAR becomes 2, 10, 50, and 100 W/kg, respectively. SAR = 0 W/kg indicates the initial state without microwave exposure

Figure 3 shows the SAR distribution, temperature elevation, and blood perfusion rate along the center axis (see Fig. 2) for peak 10 g averaged SAR values of 2, 10, 50, and 100 W/kg. The wave source was the dipole antenna. As shown in Figs. 2 and 3a–c, the penetration depth decreases as the frequency increases. As shown in Fig. 3d–f, the temperature elevation becomes larger for higher SARs. The common temperatures around the periphery of the brain (19–33 mm from the surface) are lower than the brain basal temperature of 37.3 °C. The steady-state peripheral brain temperature without exposure was 36.6 °C, which is consistent with the tendency in [42]; the peripheral temperature is approximately 0.5–0.6 °C smaller than the central temperature in the brain. The temperature around the brain surface increases and reaches around 40 °C at a peak SAR of 100 W/kg at 1 GHz. The temperature also reduces with increasing frequency.

In addition, the figures show that the elevation in skin temperature is dominant, especially at 10 GHz. As seen from Fig. 3g–i, the blood perfusion rate becomes large with the local temperature elevation. This is characterized by Eqs. 6, 7, 8, or governed by the tissue type and local temperature elevation. The brain blood perfusion rate at 1 GHz increased by 0.58, 3.1, 12.6, and 19.5% for peak SARs of 2, 10, 50, and 100 W/kg, respectively. The skin blood perfusion rate at 1 GHz increased by 4.2, 26.8, 157.8, and 309.8% for peak SARs of 2, 10, 50, and 100 W/kg, respectively. The increase in the other tissues was not notable, which is attributed to the formula we used in this study.

**Fig. 4:**
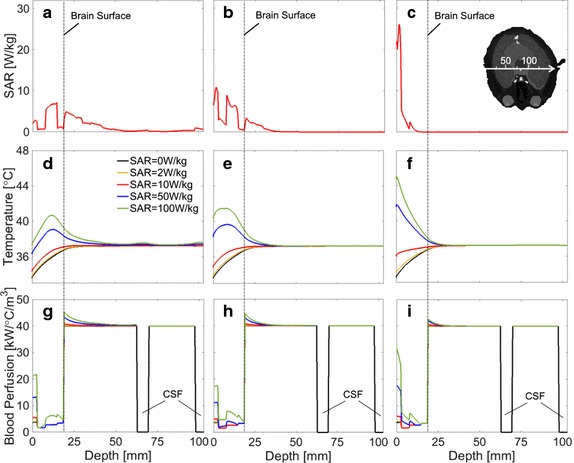
Distributions of SAR, temperature, and blood perfusion rate from the truncated plane wave. The axis passes through the cross section center in Fig. 2. SAR at **a** 1 GHz, **b** 3 GHz, and **c** 10 GHz. Temperature elevation at **d** 1 GHz, **e** 3 GHz, and **f** 10 GHz. Blood perfusion rate at **g** 1 GHz, **h** 3 GHz, and **i** 10 GHz. The incident power density was normalized so that peak SAR becomes 2, 10, 50, and 100 W/kg, respectively. SAR = 0 W/kg indicates the initial state without microwave exposure

Figure 4 shows the distribution of the SAR, temperature, and blood perfusion rate along the same axis of Fig. 3 due to plane-wave exposure. Comparing Figs. 3 and 4, the difference in the SAR distribution between the dipole antenna and the truncated plane wave is significant at 1 GHz.

### Effect of thermoregulation and heating factor

**Fig. 5:**
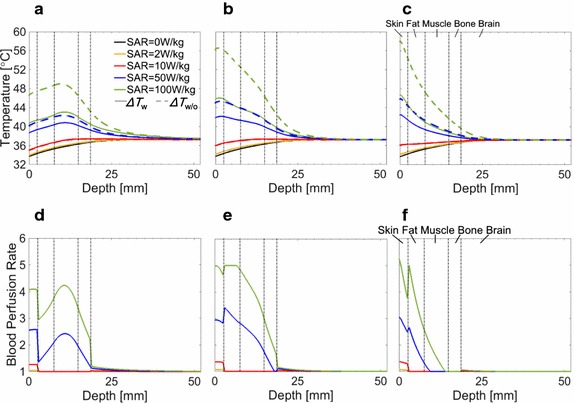
Comparison of temperature elevations with and without thermoregulation (**a**–**c**) and normalized blood perfusion (**d**–**f**). Frequencies are (**a**, **d**) 1 GHz, (**b**, **e**) 3 GHz, and (**c**, **f**) 10 GHz. The heat source is the dipole antenna. The solid line indicates temperature with thermoregulation through the cross section center in Fig. 2. (Δ*T*_*W*_ are the same data in Fig. 3). The broken line indicates the temperature without thermoregulation (Δ*T*_*W/O*_)

Figure 5a–c shows a comparison of the temperature elevation in the head with and without thermoregulation [*B*(**r**, *t*) = *B*_*0*_(**r**) in Eq. 2 over the model]. The difference in the temperature with and without thermoregulation is marginal at a peak SAR below 10 W/kg, while it is obvious at 50 and 100 W/kg. For peak SAR equal to 100 W/kg, the temperature elevation on the skin surface was 6.0, 10.6, and 11.4 °C at 1, 3, and 10 GHz, respectively. This corresponds to suppression of temperature elevation by 54.2, 53.3, and 53.6%, respectively. For 100 W/kg peak SAR, temperature elevations in the brain were suppressed by 2.5, 2.5, and 1.5 °C at 1, 3 and 10 GHz, respectively, each corresponding to a suppression of 58.9, 53.5, and 45.1%, respectively.

Figure 5d–f shows the blood perfusion rate normalized by the basal blood perfusion rate *B*_0_ for the dipole antenna. The tendency of Fig. 5a–f are similar due to an increase in blood perfusion with local temperature elevation, as shown in Eqs. 6, 7, 8. The increased blood perfusion is noticeable only in skin for a peak SAR equal to 10 W/kg. The blood perfusion increase is significant in the skin, fat, and muscle at 50 and 100 W/kg.

Table 4 shows the heating factors with thermoregulation for the peak 10 g SAR. The heating factors at 2 and 10 W/kg are relatively constant. However, they decrease by 30.6 and 52.7% at peak SARs of 50 and 100 W/kg at 10 GHz when considering thermoregulation from the dipole antenna. These results are confirmed to have a nonlinear relationship, as mentioned above.

**Table 4:** Heating factor in the brain from exposure to the dipole antenna and the truncated plane wave

	Heating factor (°C kg/W)			
	2 W/kg	10 W/kg	50 W/kg	100 W/kg
Dipole antenna				
1 GHz	0.092	0.093	0.069	0.051
3 GHz	0.072	0.073	0.053	0.038
10 GHz	0.036	0.037	0.025	0.017
Plane wave				
1 GHz	0.072	0.076	0.066	0.052
3 GHz	0.065	0.068	0.055	0.040
10 GHz	0.068	0.068	0.049	0.035

## Discussion

We computed the temperature elevation in the human head while accounting for vasodilation. Vasodilation is the most significant parameter that influences the temperature elevation due to local exposure (see also [29]). In the worst case, temperature elevation at the thermal steady state was investigated. It takes more than 30 min of constant exposure for the steady state to be reached [43]. The main contribution of this study revealed the limitation of the approach in terms of the heating factor in the regime where thermal damage may occur. Specifically, the local temperature cannot be well estimated in terms of the heating factor at the steady-state temperature. The inaccuracy arises due to ignoring thermoregulation (vasodilation).

The SAR and temperature elevation distributions were different for different wave sources, whereas the influence of internal tissues such as the brain on the temperature were marginal (Figs. 3 and 4). This is due to the heat diffusion length in biological tissue [44]. The effect of vasodilation becomes notable for tissue temperature elevations higher than 1–2 °C, which is consistent with a previous study [45]. For localized exposure in the head, averaged skin and core temperature elevations were smaller than 0.3 and 0.1 °C, respectively. Thus, thermoregulation was dominated by local temperature elevation or vasodilation. A significant difference in the temperature elevation considering thermoregulation was found in the skin and brain. In addition, for all the cases considered here, the temperature elevation was mainly induced by heat conduction of the power absorption in the outer layer of the head (skin and skull). Considering the linearity of the bioheat equation without thermoregulation [36], we can estimate the contribution of SAR in the brain to the temperature elevation in the brain. Although detailed data is not shown, its contribution at peak SAR of 2 W/kg was 25% or less at 1 GHz and becomes smaller at higher frequencies.

With increasing frequency, the SAR distributions and resulting temperature elevations were shifted to more peripheral locations in the head. At the current occupational exposure limit of 10 W/kg peak 10 g SAR in the head, the peak temperature elevation in the brain is at most 0.93 °C (see Table 2). In comparison, using a statistical approach considering more than 30 cases [38], the maximum brain temperature was observed at 1.25 °C at 10 W/kg (1.06 °C as the 95%ile value), corresponding to the maximum brain temperature of 37.8 °C. In such cases, standing wave was observed around the pinna or most energy was absorbed in the pinna. The result may change due to slight difference of the antenna position. Exposure limits for the general public are 20% of those for occupational groups, resulting in a correspondingly lower peak temperature increase at the margins of the brain.

The local brain temperature at 39–43 °C may be close to the threshold of tissue damage, however it depends on the exposure duration, etc. [46]. As shown in Table 2, the temperature may reach this 43 °C threshold at more than 100 W/kg. From the Table 2, as well as a previous study [38], the brain temperature elevation decreases as the frequency increases. The temperature elevation, however, does not decrease significantly at higher frequencies because heat is conducted away from the surface of the human head. Instead, the temperature elevation in the skin reached approximately 50 °C at 10 GHz. This is attributable to the SAR averaging algorithm. In the IEEE standard, the pinna is not included in the averaging volume. Instead, in the ICNIRP guidelines, the SAR values are averaged over single tissues that include the pinna. Thus, a straightforward comparison is not available. However, additional consideration is needed when extending SAR in a cube up to 10 GHz.

The maximum temperature elevation is in the periphery of the brain. It is generally 0.5 °C cooler than the core of the brain due to thermal interactions with the environment [42], as is apparent from Figs. 3 and 4. The peak temperature in the brain induced by RF exposure, even at occupational exposure limits, is anticipated to be comparable to the baseline temperature in the core of the brain. The peak temperature elevation in the brain at exposure limits for the general public are anticipated to be well within the diurnal variation in brain temperature (about 1 °C peak to peak) [47].

As shown in Fig. 5, the temperature elevation reduced significantly due to the increased blood perfusion rate in the skin and muscle. In contrast, the increase of blood perfusion in the brain was marginal. As mentioned above, the SAR in the brain contributes slightly to the brain temperature elevation, and the suppressed brain temperature with thermoregulation mainly is attributable to thermoregulation in the surface tissues.

The main limitation of this study is that thermoregulation modeling was based on animal studies [23]. It is difficult to determine the similarity of the thermoregulation details between humans and rats or how the results can be extrapolated to humans. However, human thermoregulation is known to be superior to that of other species, especially rodents [21]. Thus, the actual temperature elevation is expected to be lower in healthy young adults, as thermoregulation in the elderly becomes weak because of degradation of the periphery sensor. Moreover, Philips et al. [48] noted the vasomotor is positively correlated with body mass. Gordon et al. [21] explained that even though there is a large difference in the body masses between human and rat, the values of the different thermoregulatory variables are very close. In fact, our computational modeling has been demonstrated to be useful for human leg exposure to hot water (42 °C) [22]. In addition, we reviewed the thermoregulation modeling and compared our results to their performance for the whole-body exposures [45]. The validation for vasodilation localized microwave exposure discussed for only rats [23] and rabbit [12]. The exposure scenario is not feasible for invasive temperature measurement in the human.

Another factor that can influence our results is the initial blood perfusion rate. We computed the temperature elevation using the basal blood perfusion in McIntosh et al. [49, 50]. The maximum temperature elevations in the brain due to dipole antenna exposure at 1 GHz appeared gray matter; these values were 3.19 and 4.76 °C for peak SAR values of 50 and 100 W/kg, respectively. The difference was less than 10%. The difference of basal blood perfusion may affect the temperature in white matter. The temperature elevation is approximately estimated by volume averaged blood flow as in [34]. Thus, the value of our skin blood is different from the value in the review, but the difference in the computed temperature was at most 25% [51].

We chose a model of inner tissue where the vasodilation works only at 39 °C (Eq. 6) for more strict temperature elevation. However, a recent study suggested the possibility of working with smaller temperature elevations (estimation to the muscle and fat) [52]. If we used such a model, the temperature elevation reduces by 20% or more at higher whole-body SAR [45]. Thus, the actual temperature elevation would be even lower in healthy young adults. As in [52], the parameters are not well validated by the blood flow measurement and thus further discussion is not given in this study.

Head models commonly used in dosimetry have a less detailed structure of the brain. There are many tissues between the skull itself and the brain in a real head (e.g., superior cerebral veins). The vasculature is considered to be a source of heat transfer [53], which is not considered in this study. Due to the finite resolution of the anatomical models, the anatomy of dura and cerebrospinal fluid etc. cannot be modeled accurately. The temperature elevation would be different between a plane wave applied to the whole body and the truncated head model.

Finally, if the heating factor is used to set the limit in the international standards, the location of the maximum temperature elevation is not considered. It is the nature of electromagnetic fields to decay exponentially from the body surface, especially for near field exposures from portable devices (see Fig. 2). Thus, the limit derived based on the heating factor may provide conservative limits. Instead, this study showed that the effect of thermoregulation is not significant for a local temperature elevation of ~ 1 °C because thermoregulation has not been activated.

## Conclusions

This study investigated temperature elevations in the brain considering vasodilation for microwave exposure at the limits prescribed in the international guidelines/standards. We computationally estimated the temperature elevation in the brain for excessive SAR. The current limit of 10 W/kg has the margin of more than 10 when compared to the thermal damage of threshold [46]. This margin is caused by thermoregulation and is not be expected from the linear model that ignores thermoregulation. Note that damage is characterized by the duration of the exposure, which has not been significantly addressed in this paper. Temperature elevation in the brain at the current guideline limit for occupational exposure (i.e., 10 W/kg averaged over 10 g) was comparable to the variability from daily environmental changes and/or diurnal changes.

## References

[1] International Commission on Non-Ionizing Radiation Protection (ICNIRP) (1998). Guidelines for limiting exposure to time-varying electric, magnetic, and electromagnetic fields (up to 300 GHz). Health Phys.

[2] IEEE C95.1. IEEE standard for safety levels with respect to human exposure to radio frequency electromagnetic fields, 3 kHz to 300 GHz. IEEE Std C95.1-2005 (Revision IEEE Std C95.1-1991). 2006.

[3] Matthes R (1998). Response to questions and comments on ICNIRP. Health Phys.

[4] International Electrotechnical Commission. Human exposure to radio frequency fields from hand-held and body-mounted wireless communication devices—human models, instrumentation, and procedures. Part 1. 2010;IEC 62209-2.

[5] IEEE Standard C95.3. IEEE recommended practice for measurements and computations of radio frequency electromagnetic fields with respect to human exposure to such fields, 100 kHz–300 GHz. Institute of Electrical and Electronics Engineers; 2002.

[6] Hirata A, Fujiwara O (2009). The correlation between mass-averaged SAR and temperature elevation in the human head model exposed to RF near-fields from 1 to 6 GHz. Phys Med Biol.

[7] Razmadze A, Shoshiashvili L, Kakulia D, Zaridze R, Bit-Babik G, Faraone A (2009). Influence of specific absorption rate averaging schemes on correlation between mass-averaged specific absorption rate and temperature rise. Electromagnetics.

[8] McIntosh RL, Anderson V (2010). SAR versus sinc: what is the appropriate RF exposure metric in the range 1–10 GHz? Part II: using complex human body models. Bioelectromagnetics.

[9] WHO. WHO research agenda for radiofrequency fields. Geneva: World Health Organisation; 2010. http://apps.who.int/iris/bitstream/10665/44396/1/9789241599948_eng.pdf of subordinate document. Accessed 28 Oct 2017.

[10] Guy AW, Lin JC, Kramar PO, Emery AF (1975). Effect of 2450-MHz radiation on the rabbit eye. IEEE Trans Microw Theory Tech.

[11] Kojima M, Hata I, Wake K, Watanabe S, Yamanaka Y, Kamimura Y (2004). Influence of anesthesia on ocular effects and temperature in rabbit eyes exposed to microwaves. Bioelectromagnetics.

[12] Hirata A, Watanabe S, Kojima M, Hata I, Wake K, Taki M (2006). Computational verification of anesthesia effect on temperature variations in rabbit eyes exposed to 2.45 GHz microwave energy. Bioelectromagnetics.

[13] Hirata A, Masuda H, Kanai Y, Asai R, Fujiwara O, Arima T (2011). Computational modeling of temperature elevation and thermoregulatory response in the brains of anesthetized rats locally exposed at 1.5 GHz. Phys Med Biol.

[14] Bernardi P, Cavagnaro M, Pisa S, Piuzzi E (2003). Specific absorption rate and temperature elevation in a subject exposed in the far-field of radio-frequency sources operating in the 10–900-MHz range. IEEE Trans Biomed Eng.

[15] Foster KR, Adair ER (2004). Modeling thermal responses in human subjects following extended exposure to radiofrequency energy. Biomed Eng Online.

[16] Hirata A, Asano T, Fujiwara O (2007). FDTD analysis of human body-core temperature elevation due to RF far-field energy prescribed in the ICNIRP guidelines. Phys Med Biol.

[17] Moore SM, McIntosh RL, Iskra S, Wood AW (2015). Modeling the effect of adverse environmental conditions and clothing on temperature rise in a human body exposed to radio frequency electromagnetic fields. IEEE Trans Biomed Eng.

[18] Moore SM, McIntosh RL, Iskra S, Lajevardipour A, Wood AW (2017). Effect of adverse environmental conditions and protective clothing on temperature rise in a human body exposed to radiofrequency electromagnetic fields. Bioelectromagnetics.

[19] Wainwright PR (2007). Computational modelling of temperature rises in the eye in the near field of radiofrequency sources at 380, 900 and 1800 MHz. Phys Med Biol.

[20] Masuda H, Hirata A, Kawai H, Wake K, Watanabe S, Arima T (2011). Local exposure of the rat cortex to radiofrequency electromagnetic fields increases local cerebral blood flow along with temperature. J Appl Physiol.

[21] Gordon CJ (1993). Temperature regulation in laboratory rodents.

[22] Hirata A, Nomura T, Laakso I (2015). Computational estimation of body temperature and sweating in the aged during passive heat exposure. Int J Therm Sci.

[23] Kodera S, Gomez-Tames J, Hirata A, Masuda H, Arima T, Watanabe S (2017). Multiphysics and thermal response models to improve accuracy of local temperature estimation in rat cortex under microwave exposure. Int J Environ Res Public Health.

[24] Stolwijk JA. A mathematical model of physiological temperature regulation in man. NASA Contract. Rep. 1971;CR-1855:77.

[25] Hirata A, Asano T, Fujiwara O (2008). FDTD analysis of body-core temperature elevation in children and adults for whole-body exposure. Phys Med Biol.

[26] Taflove A, Hagness SC (2005). Computational electrodynamics: the finite-difference time-domain method.

[27] Gabriel C. Compilation of the dielectric properties of body tissues at RF and microwave frequencies. Environ Health. 1996; Report No. 21.

[28] Roden AJ, Gedney SD (2000). Convolutional PML (CPML): an efficient FDTD implementation of the CFS-PML for arbitrary media. Microw Opt Technol Lett.

[29] Hirata A, Fujimoto M, Asano T, Wang J, Fujiwara O, Shiozawa T (2006). Correlation between maximum temperature increase and peak SAR with different average schemes and masses. IEEE Trans Electromagn Compat.

[30] Pennes HH (1948). Analysis of tissue and arterial blood temperatures in the resting human forearm. J Appl Physiol.

[31] Hirata A, Fujiwara O (2009). Modeling time variation of blood temperature in a bioheat equation and its application to temperature analysis due to RF exposure. Phys Med Biol.

[32] Hoque M, Gandhi OP (1988). Temperature distributions in the human leg for VLF-VHF exposures at the ANSI-recommended safety levels. IEEE Trans Biomed Eng.

[33] Chatterjee I, Gandhi OP (1983). An inhomogeneous thermal block model of man for the electromagnetic environment. IEEE Trans Biomed Eng.

[34] Hirata A, Fujiwara O, Shiozawa T (2006). Correlation between peak spatial-average SAR and temperature increase due to antennas attached to human trunk. IEEE Trans Biomed Eng.

[35] Hirata A, Watanabe S, Fujiwara O, Kojima M, Sasaki K, Shiozawa T (2007). Temperature elevation in the eye of anatomically based human head models for plane-wave exposures. Phys Med Biol.

[36] Fiala D, Lomas K, Stohrer M (2001). Computer prediction of human thermoregulatory and temperature responses to a wide range of environmental conditions. Int J Biometeorol.

[37] Nagaoka T, Watanabe S, Sakurai K, Kunieda E, Watanabe S, Taki M (2004). Development of realistic high-resolution whole-body voxel models of Japanese adult males and females of average height and weight, and application of models to radio-frequency electromagnetic-field dosimetry. Phys Med Biol.

[38] Morimoto R, Laakso I, De Santis V, Hirata A (2016). Relationship between peak spatial-averaged specific absorption rate and peak temperature elevation in human head in frequency range of 1–30 GHz. Phys Med Biol.

[39] Hashimoto Y, Hirata A, Morimoto R, Aonuma S, Laakso I, Jokela K (2017). On the averaging area for incident power density for human exposure limits at frequencies over 6 GHz. Phys Med Biol.

[40] Foster KR, Ziskin MC, Balzano Q, Bit-Babik G. Modeling tissue heating from exposure to radiofrequency energy and its relevance to exposure limits: heating factor. Health Phys J (in press). 10.1097/HP.0000000000000854 29957690

[41] Dielectric properties of body tissues. http://niremf.ifac.cnr.it/tissprop/. Accessed 29 Oct 2017.

[42] Wang H, Wang B, Normoyle KP, Jackson K, Spitler K, Sharrock M (2014). Brain temperature and its fundamental properties: a review for clinical neuroscientists. Front Neurosci.

[43] Morimoto R, Hirata A, Laakso I, Ziskin MC, Foster KR (2017). Time constants for temperature elevation in human models exposed to dipole antennas and beams in the frequency range from 1 to 30 GHz. Phys Med Biol.

[44] Foster KR, Ziskin MC, Balzano Q (2016). Thermal response of human skin to microwave energy: a critical review. Health Phys.

[45] Laakso I, Hirata A (2011). Dominant factors affecting temperature rise in simulations of human thermoregulation during RF exposure. Phys Med Biol.

[46] Van Rhoon GC, Samaras T, Yarmolenko PS, Dewhirst MW, Neufeld E, Kuster N (2013). CEM43 °C thermal dose thresholds: a potential guide for magnetic resonance radiofrequency exposure levels?. Eur Radiol.

[47] Aschoff J (1983). Circadian control of body temperature. J Therm Biol.

[48] Phillips PK, Heath JE (1995). Dependency of surface temperature regulation on body size in terrestrial mammals. J Therm Biol.

[49] McIntosh RL, Anderson V (2010). A comprehensive tissue properties database provided for the thermal assessment of a human at rest. Biophys Rev Lett.

[50] McIntosh RL, Anderson V (2010). Erratum: a comprehensive tissue properties database provided for the thermal assessment of a human at rest. Biophys Rev Lett.

[51] Laakso I, Morimoto R, Hirata A, Member S (2016). Computational dosimetry of the human head exposed to near-field microwaves using measured blood flow. IEEE Trans Electromagn Compat.

[52] Murbach M, Neufeld E, Capstick M, Kainz W, Brunner DO, Samaras T (2014). Thermal tissue damage model analyzed for different whole-body SAR and scan durations for standard MR body coils. Magn Reson Med.

[53] Kotte A, Van Leeuwen G, Bree D (1999). Modelling the thermal impact of a discrete vessel tree. Phys Med Biol.

